# Sick leave in early axial spondyloarthritis: the role of clinical and socioeconomic factors. Five-year data from the DESIR cohort

**DOI:** 10.1136/rmdopen-2021-001685

**Published:** 2021-06-25

**Authors:** Elena Nikiphorou, Pedro D Carvalho, Annelies Boonen, Bruno Fautrel, Pascal Richette, Pedro M Machado, Desirée van der Heijde, Robert Landewé, Sofia Ramiro

**Affiliations:** 1 Leiden University Medical Center (LUMC), Department of Rheumatology, Leiden, The Netherlands; 2 Centre for Rheumatic Diseases, King's College London, London, UK; 3 Department of Rheumatology, King’s College Hospital, London, UK; 4 Department of Rheumatology, Centro Hospitalar Universitário do Algarve, Faro, Portugal; 5 Lisbon Academic Medical Centre, Lisbon, Portugal; 6 Care and Public Health Research Institute (CAPHRI), Maastricht University, Maastricht, The Netherlands; 7 Maastricht University Medical Center, Department of Internal Medicine, Division of Rheumatology, Maastricht, The Netherlands; 8 Sorbonne University – Assistance Publique Hopitaux de Paris, Pitie Salpetriere Hospital, Dept of Rheumatology. Pierre Louis Institute for Epidemiology and Public Health, INSERM UMRS 1136, PEPITES Teams, Paris, France; 9 Université de Paris, Hopital Lariboisière, Department of Rheumatology, INSERM U1132, Paris, France; 10 Centre for Rheumatology & Department of Neuromuscular Diseases, University College London, London, UK; 11 National Institute for Health Research (NIHR) Biomedical Research Centre, University College London Hospitals NHS Foundation Trust, London, UK; 12 Department of Rheumatology, Northwick Park Hospital, London North West University Healthcare NHS Trust, London, UK; 13 Zuyderland Medical Center, Department of Rheumatology, Heerlen, The Netherlands; 14 Amsterdam University Medical Center, Amsterdam, The Netherlands

**Keywords:** epidemiology, inflammation, spondylitis, ankylosing

## Abstract

**Objectives:**

To investigate the occurrence of sick leave (SL) and the impact of clinical and socioeconomic factors on SL in early axial spondyloarthritis (axSpA).

**Methods:**

Patients with a clinical diagnosis of axSpA from the DEvenir des Spondyloarthrites Indifférenciées Récentes (DESIR) cohort with work-related data and up to 5-year follow-up were studied. Incidence, time to first SL and potential role of baseline and time-varying clinical and socioeconomic factors (age, gender, ethnicity, education, job type, marital and parental status) were analysed. Univariable analyses, followed by collinearity and interaction tests, guided subsequent multivariable time-varying Cox survival model building.

**Results:**

In total, 704 axSpA patients were included (mean (SD) age 33.8 (8.6); 46% men). At baseline, 80% of patients were employed; of these, 5.7% reported being on SL. The incidence of SL among those at risk during the study period (n=620, 88%) was 0.05 (95% CI 0.03 to 0.06) per 1000 days of follow-up. Mean (SD) time to first SL was 806 (595) days (range: 175–2021 days). In multivariable models, male gender (HR 0.41 (95% CI 0.20 to 0.86)) and higher education (HR 0.48 (95% CI 0.24 to 0.95)) were associated with lower hazard of SL, while higher disease activity (HR 1.49 (95% CI 1.04 to 2.13)), older age, smoking and use of tumour necrosis factor inhibitors were associated with higher hazard of SL.

**Conclusions:**

In this early axSpA cohort of young, working-age individuals, male gender and higher education were independently associated with a lower hazard of SL, whereas older age and higher disease activity were associated with higher hazard of SL. The findings suggest a role of socioeconomic factors in adverse work outcomes, alongside active disease.

**Key messages**

**What is already known about this subject?**

- Data on sick leave as an adverse work outcome in axial spondyloarthritis (axSpA) are sparse and generally limited to established radiographic axSpA.

**What does this study add?**

- With a focus on socioeconomic factors, this study identifies personal contextual factors such as lower education, older age and female gender to be independently associated with a higher hazard of sick leave.
- This study provides evidence for the association between high disease activity and sick leave in early axSpA.

**How might this impact on clinical practice or further developments?**

- In early axSpA, older age, female gender and lower educational attainment are associated with higher sick leave and should therefore be considered when tailoring care and supporting individuals in their work role.
- From the early stages of the disease, it is important to consider personal contextual factors and control disease activity in an attempt to avoid sick leave.

## Introduction

Axial spondyloarthritis (axSpA) is a disease of young individuals, typically of working age.^1^ Studies to date suggest substantial consequences of disease on work-related outcomes. Many studies, however, have tended to focus on established disease and on a more permanent adverse outcome, work disability.^2–4^ Even more important than work disability is perhaps thinking of prequels to this often irreversible outcome, such as presenteeism and sick leave (SL).^5–9^

The findings of studies to date that have sought to examine specifically SL, vary widely in the literature and across countries.^10–12^ SL is to a large extent an individual’s decision and also driven by the social security system of a country, aside from a disease-driven outcome; in this regard, personal contextual factors could be implicated making it, in this regard, particularly attractive. Some studies suggest significant rates of SL, much higher than the general population.^13^ Others suggest rates of SL similar to the general population.^14^ Such discrepancies stem partly from methodological challenges, including small sample sizes, lack of clarity and/or consistency in the way SL is reported and importantly from the focus being on longstanding radiographic axSpA (r-axSpA).^15^ Clinical factors such as high disease activity and decreased physical functioning have been linked to adverse work outcomes including work productivity loss and SL in axSpA.^10 11 16 17^ In support of this notion, use of tumour necrosis factor inhibitors (TNFi) has been associated with significant reductions in SL.^18^

Studies in longstanding r-axSpA suggest that lower social class as reflected by lower educational status, manual/physically demanding jobs, associate with unfavourable work outcomes.^12^ Yet, the effects of these factors specifically on SL have not widely been studied. Based on longitudinal data from the Outcome in Ankylosing Spondylitis International Study (OASIS), disease activity and physical function predicted first and recurrent SL to some extent, supporting the notion that worse disease leads to more SL, but this was only observed in patients with low educational attainment.^17^ Again, these observations were in established disease (16 years symptom duration on average) limiting the generalisability of the results to patients with early disease.

Acknowledging the general lack of data in early disease and using one of the most well-established early axSpA cohorts in Europe, the DEvenir des Spondyloarthrites Indifférenciées Récentes (DESIR) cohort, we wished to explore the occurrence of SL and the impact of clinical and socioeconomic factors on SL.

## Methods

### Study population

Data from the French prospective, nationwide, multicentre (n=25 centres) DESIR cohort (clinicaltrials.gov ID: NCT01648907) were used.^19^ The cohort included consecutive patients with inflammatory back pain lasting ≥3 months but <3 years, and with a clinical diagnosis of axSpA according to their rheumatologist. Patients with work-related data were identified as the ‘study population’ and included patients who had reported the outcome of interest (SL) also at entry into the cohort. Acknowledging variation in the evolution of disease over time, rheumatologists were asked to provide a level of confidence when formulating a diagnosis of SpA, with a cut-off of ≥5 out of 10 indicating confidence in the diagnosis. By the end of the 5-year follow-up, for 47 (6.6%) patients in DESIR, an alternative diagnosis became apparent and they were excluded from the follow-up of the cohort.^20^ Being a small group and while at the same time wishing to have a pragmatic, real-life approach when analysing DESIR, it was considered appropriate to not exclude this group from our study.

Free-text information provided in the case-reporting form (CRF) was also used where available to supplement the information and help group patients into working and non-working categories. Written informed consent was obtained from participating patients before inclusion into the study.

### Sick leave

Information on SL was based on self-report and obtained every 6 months during the first 2 years of follow-up and yearly thereafter via three main routes: (a) questions where the patients were asked to report that they were on SL; (b) questions asking for a date of SL; (c) text information provided by patients including reasons for not working.

A patient was categorised as being in ‘first sick leave’ from the time point for which they first reported SL, until the last time point on which they reported SL and the date that was given was the same as (or within one month of) the date they reported for their first SL. A patient was categorised as being in ‘recurrent sick leave’ if they reported SL and a date was given that was more than one month after the date they reported for their first SL.

Patient responses varied and discrepancies were noted in the way patients completed the relevant questions on SL and date for this. For example, despite a record of SL, a date was not always provided and vice versa. When a patient reported SL as the reason for not working but did not provide a date in the relevant question on the CRF for SL, the date of SL was imputed as the mid-date between the current date of consultation (at which they reported being on SL) and their previous date of consultation. Patients retired, those with work disability and those with a date of SL prior to their baseline date of consultation, were considered as not ‘at-risk’ for the SL outcome. They may have though contributed to the analyses of SL over time during the period they were ‘at risk’, for example, when work disability occurred during the follow-up. Inaccuracies in the dates of SL reported and also its duration (eg, reported as zero days despite SL being indicated, or sometimes reported as 365 days, while these cannot correspond to the number of working days lost, among other) prompted a restriction in the analysis to factors associated with the first episode of SL (see the Statistical analysis section).

### Independent variables

Socioeconomic variables were among the main independent variables of interest, along with clinical variables. The majority of variables were assessed at the same time points as those of SL; some only at baseline (see below). Variables were used in their time-varying form (ie, at every follow-up, allowing them to vary over time) where appropriate to ensure optimal use of the data available.

#### Socioeconomic variables

Socioeconomic variables included age, gender, educational status (low (primary or secondary education) vs high (university education)), ethnicity (Caucasian vs other), job type (blue-collar (manual labour work) vs white-collar (sedentary, office-based work)), marital status (married/in couple vs not) and parental status (number of children), included in their time-varying form.

#### Clinical variables

Clinical outcomes of interest included measures of disease activity, function and spinal mobility, all included in their time-varying form. For disease activity, the Ankylosing Spondylitis Disease Activity Score (ASDAS) with C reactive protein (CRP) and the Bath Ankylosing Spondylitis Disease Activity Index (BASDAI) were used. Laboratory measures of inflammation included CRP measured in mg/L in its continuous form, as well as in a binary form of the variable to indicate raised (>6 mg/L) versus non-raised CRP at every visit. Measures of physical function included the Bath Ankylosing Spondylitis Functional Index (BASFI) and for mobility, the Bath Ankylosing Spondylitis Metrology Index (BASMI).

#### Other variables

Other variables included in the analysis in their time-varying form, included smoking (current vs non-current smoker since last visit), history of extra-musculoskeletal manifestations, namely uveitis, psoriasis and inflammatory bowel disease (IBD). Disease characteristics recorded at entry into the study and used in their baseline form, included: symptom duration studied as a continuous variable; presence of Human Leukocyte Antigen B27 (HLA-B27) and hip involvement. Imaging outcomes studied included sacroiliac joints (SIJ) radiographs using the modified New York grading (mNY, 0–8)^21^ and MRI-SIJ using the Spondyloarthritis Research Consortium of Canada scoring system (SPARCC, 0–72).^22^

Comorbidity burden was reflected in a comorbidity ‘count’ variable that was computed to include the following comorbidities: chronic pulmonary disease, ischaemic heart disease, pericarditis, heart failure, cardiac valve disease including aortic insufficiency, heart rhythm disorders, hypertension, cerebrovascular accidents, diabetes, gastric ulcers/perforation/haemorrhage, lymphoproliferative disease, organ neoplastic disease, depression/anxiety (using the Short Form-36 Mental Component Score; threshold of ≤38 to identify the presence of either depression or anxiety).^23^ The higher the count, the higher the number of comorbidities in an individual.

Treatment variables were analysed in their time-varying form: non-steroidal anti-inflammatory drugs (NSAIDs) based on computation of the Assessment of SpondyloArthritis international Society (ASAS) NSAID Score (0–400),^24^ also tested as binary variable (NSAID use in the last 6 months); conventional synthetic disease-modifying anti-rheumatic drug use; steroid use and TNFi use.

### Statistical analysis

Descriptive statistics for baseline characteristics of patients in the study population and those ‘at-risk for sick leave’ at any point during follow-up were applied. The incidence rate of SL from those ‘at-risk’ of the outcome (SL) among the total study population was calculated based on the total at-risk observation time, in days. In other words, this was the sum of the days that each patient was at risk (from entering the at-risk period to the end of follow-up or to their failure, ie, reaching SL, being censored due to loss-to-follow or no longer being at risk (eg, work disability, retired or with a date of SL prior or equal to the baseline date of consultation) or reaching the 5-year follow-up).

Patients who at any point during the at-risk period developed SL (‘Ever sick leave’) were compared with those who did not report SL during the at-risk observation period (‘Never sick leave’). These groups excluded patients reporting SL at baseline and those retired. Comparisons between the ‘Ever sick leave’ versus the ‘Never sick leave’ categories were undertaken using the Wilcoxon test for continuous variables (skewed distribution) and either the χ^2^ or Fisher’s exact test for categorical variables.

Time-varying cox survival analysis was used to study time to first SL for all patients who were at risk of SL at any time point during the 5 years of DESIR. A patient was considered a ‘failure’ at the date of consultation at which they first reported SL. The multivariable model analyses were guided by relevant interaction analyses, namely between disease activity (ASDAS) or function (BASFI) and each of the following: age, gender and education. Where statistically relevant interactions (p<0.150) were identified, model stratification was performed to assess the clinical relevance. A multistep technique was followed to identify the most parsimonious, multivariable models for the impact of clinical and socioeconomic factors on SL. First, univariable analysis was undertaken with SL as the dependent outcome. Variables with a p<0.20 were subsequently tested in stepwise forward Cox regression models. Socioeconomic and clinical variables were entered first as main variables of interest. Variables were retained in the models if significant at the p<0.05 level or if identified as confounders of the socioeconomic variables (resulting in a change of the HR by >15%). Collinearity checks were also undertaken between individual variables, followed by testing of the final models for multicollinearity and violation of proportional hazards. Separate models have been constructed with the main clinical variables (ASDAS, BASFI and BASMI) where necessary, to allow for the individual effects of these variables to be explored.

Sensitivity analyses were performed in the subgroup of patients fulfilling the ASAS classification criteria,^25^ following each of the above analysis steps.

## Results

### Baseline characteristics

A total of 704 (99%) patients in DESIR had provided information on work-related data and could be studied. At baseline, the mean (SD) age of the eligible study population was 33.8 (8.6), with 46% being men and 90% of Caucasian ethnicity. Higher (university) education was attained by 59%. At baseline, mean (SD) ASDAS was 2.7 (0.9), BASFI 3.0 (2.3) and BASMI 2.4 (1.0). The study population was categorised into patients who were in employment (including those on SL), a total of 561 patients (80%), or unemployed (including those on work disability, retired, housewife/houseman, student in training, other). Baseline characteristics for the study population are shown in online supplemental table S1. There were no patients on TNFi at baseline, although over time there was an increasing proportion of people on the drugs with 40% reporting use of TNFi at the 5-year time point, accounting for those remaining in follow-up.

10.1136/rmdopen-2021-001685.supp1 Supplementary data

### Risk of SL

**Figure 1:**
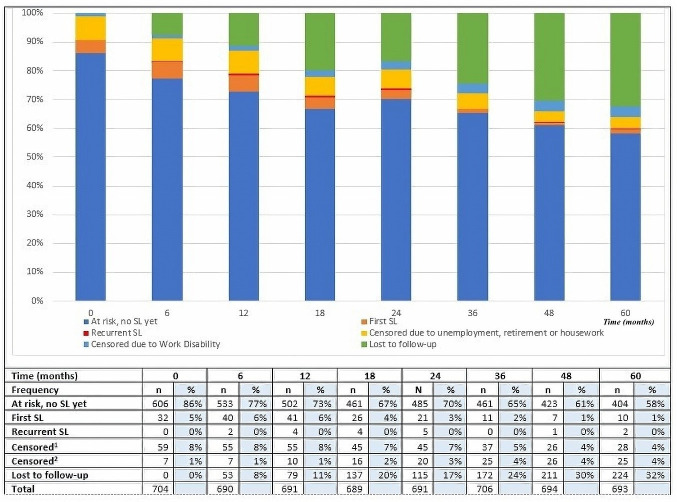
Distribution of first and recurrent sick leave episodes over time in the study population. SL, sick leave. ^1^Censored due to unemployment/retirement/housework. ^2^Censored due to work disability.

Of 561 working at baseline, 5.7% (n=32) reported being on SL already at study baseline. Of the total study population, 88% (n=620) were at risk of a future episode of SL over the study period and were included in the time-to-event analyses of SL. Patients reported being in and out of SL at various time points throughout the study. The distribution of first and recurrent SL episodes over the 5 years of follow-up is shown in figure 1.

In total, 43 (7%) of patients reported SL for the first time during the 5 years of follow-up (ie, a new episode, not counting the patients already with SL at baseline), with the mean (SD) time to SL being 806 (595) days, minimum 175 days, maximum 2021 days (5.5 years). A first SL was reported by 25% of patients at 364 days of follow-up: by 50% and 75% at 545 and 1172 days, respectively. The SL incidence rate across the study follow-up and among those at risk of SL was 0.05 (95% CI 0.03 to 0.06) per 1000 days calculated in a total person-days of observation of 913 559. The latter represents the total time (in days) of all patients (n=620) at risk for SL (from entering the at-risk period to the end of follow-up or to the first ‘failure’).

Significant differences were noted in the baseline characteristics of patients who reported SL at any point during the study (‘Ever sick leave’) compared with those who never did (‘Never sick leave’). This was notable for socioeconomic factors where for example older age, more females, lower education, being in married/couple relationship were seen in those who were ‘Ever’ on SL. Similarly, higher disease activity and more use of NSAIDs were seen in those in the ‘Ever sick leave’ group (table 1). Although TNFi were not used at baseline, the use of these agents over the 5 years was significantly different between the ‘Ever’ versus ‘Never’ sick leave groups, with 61% using a TNFi in the former group, compared with 35% in the latter group (p=0.001).

**Table 1:** Baseline characteristics of patients at risk of sick leave and for those with ‘Ever’ and ‘Never’ sick leave during the 5-year follow-up

Baseline variables	At risk of sick leave	Ever sick leave	Never sick leave	P value*
	Total N=620	Total N=43	Total N=577	
Age, years	33.4 (8.6)	36.8 (8.3)	33.2 (8.6)	0.007
Male gender	288, 47%	11, 26%	277, 48%	0.004
Caucasian ethnicity	561, 91%	38, 88%	523, 91%	0.625
Higher education	387, 63%	16, 38%	371, 64%	0.001
Missing values (%)	2 (0.3)	1 (2.3)	1 (0.2)	
Blue-collar profession	88, 16%	7, 18%	81, 16%	0.839
Missing values (%)	82 (13.2)	3 (7.0)	79 (13.7)	
Married/In couple	388, 63%	33, 81%	355, 62%	0.016
Missing values (%)	3 (0.5)	2 (4.6)	1 (0.2)	
Parental status, number of children				0.254
0	272, 45%	13, 33%	259, 46%	
1	111, 18%	12, 30%	99, 18%	
2	148, 25%	12, 30%	136, 24%	
3	57, 9%	2, 5%	55, 10%	
4	9, 1%	1, 3%	8, 1%	
5	6, 1%	–	6, 1%	
6	1, 0%	–	1, 0%	
Missing values (%)	16 (2.6)	3 (7.0)	13 (2.3)	
Smoking, current	217, 35%	24, 56%	193, 34%	0.003
Missing values (%)	4 (0.6)	0 (0.0)	4 (0.7)	
HLA-B27 positivity	368, 60%	20, 47%	348, 60%	0.073
Missing values (%)	1 (0.2)	0 (0.0)	1 (0.2)	
Symptom duration, years	1.5 (0.9)	1.6 (0.9)	1.5 (0.9)	0.420
Missing values (%)	1 (0.2)	0 (0.0)	1 (0.2)	
ASDAS-CRP	2.6 (0.9)	2.8 (0.8)	2.6 (0.9)	0.064
Missing values (%)	28 (4.5)	4 (9.3)	24 (5.2)	
Elevated CRP (>6 mg/L)	163, 27%	10, 24%	153, 27%	0.612
Missing values (%)	20 (3.2)	1 (2.3)	19 (3.3)	
CRP, mg/L	7.6 (13.5)	5.9 (8.6)	7.8 (13.8)	0.338
Missing values (%)	20 (3.2)	1 (2.3)	19 (3.3)	
BASDAI, 0–10	4.3 (2.0)	5.0 (2.0)	4.3 (2.0)	0.009
Missing values (%)	3 (0.5)	2 (4.7)	1 (0.2)	
BASFI, 0–10	2.9 (2.2)	3.8 (2.2)	2.8 (2.2)	0.003
Missing values (%)	6 (1.0)	1 (2.3)	5 (0.9)	
BASMI, 0–10	2.4 (0.9)	2.8 (0.9)	2.4 (0.9)	0.001
Missing values (%)	53 (8.5)	3 (7.0)	50 (8.7)	
History of uveitis	57, 9%	2, 5%	55, 10%	0.285
History of psoriasis	109, 18%	7, 16%	102, 18%	0.816
History of IBD	32, 5%	2, 5%	30, 5%	0.875
History of peripheral arthritis	40, 7%	3, 7%	37, 6%	0.887
Missing values (%)	1 (0.2)	0 (0.0)	1 (0.2)	
Comorbidity count, 0–4	0.6 (0.6)	0.7 (0.5)	0.6 (0.6)	0.190
Missing values (%)	9 (1.5)	3 (6.8)	6 (1.0)	
SIJ MRI, SPARCC score, 0–72	3.1 (6.6)	3.1 (6.6)	3.1 (6.6)	0.997
Missing values (%)	22 (3.5)	3 (7.0)	19 (3.3)	
SIJ radiographs, mNY grading, 0–8	1.3 (1.6)	1.1 (1.4)	0.3 (1.7)	0.695
Missing values (%)	19 (3.1)	1 (2.3)	18 (3.1)	
NSAID score in last week, 0–400	56.3 (52.3)	73.5 (53.0) 1 (2.3)	56.3 (52.3) 11 (1.9)	0.021
Steroid use	72, 12%	6, 14%	66, 11%	0.619
TNFi use	0, 0%	0, 0%	0, 0%	–

Mean (SD) for continuous variables; n, % for categorical variables. N number reported where there were missing data for the specific variable.

*Comparisons between the Ever sick leave versus the Never sick leave categories were undertaken using the Wilcoxon test for continuous variables and either the χ^2^ or Fisher's exact test for categorical variables.

ASDAS, Ankylosing Spondylitis Disease Activity Score; BASDAI, Bath Ankylosing Spondylitis Disease Activity Index; BASFI, Bath Ankylosing Spondylitis Functional Index; BASMI, Bath Ankylosing Spondylitis Metrology Index; CRP, C reactive protein; HLA-B27, Human Leukocyte Antigen B27; IBD, inflammatory bowel disease; mNY, modified New York; NSAIDs, non-steroidal anti-inflammatory drugs; SIJ, sacroiliac joints; SPARCC, Spondyloarthritis Research Consortium of Canada scoring system; TNFi, tumour necrosis factor inhibitor.

### Effect of socioeconomic factors and clinical variables on SL

No clinically relevant interactions were identified that necessitated model stratification. Several clinical and socioeconomic variables were univariably associated with SL; for instance, higher education was associated with a lower hazard of SL with a HR of 0.33 (95% CI 0.17 to 0.61) (online supplemental table S2).

In multivariable models, time-varying higher disease activity was associated with more SL. Specifically, every unit increase in ASDAS was associated with a 49% increase in the hazard of SL. Similarly, older age, smoking and use of TNFi, were associated with a higher hazard of SL. In separate models and unlike disease activity, functional ability (BASFI) and spinal mobility (BASMI) were not significantly independently predictive of SL. Across all models, male gender and higher education were associated with a lower hazard of SL: in the ASDAS model, with a 59% (HR 0.41, 95% CI 0.20 to 0.86) and 52% (HR 0.48, 95% CI 0.24 to 0.95) decrease in the hazard of SL, respectively. Smoking strongly associated with a higher hazard of SL (HR >2.5) across all models. The use of TNFi was significantly associated with a higher hazard of SL and in the case of the BASFI model, steroid use was also significantly associated with a higher hazard of SL. The effect of education in the main model is also graphically shown in online supplemental figure S1. Table 2 shows the most parsimonious models with each of the three clinical variables (ASDAS, BASFI and BASMI) tested in separate models and allowing for their separate effects to be seen. BASDAI and BASDAI/CRP in models in the place of ASDAS to explore if a patient reported outcome had a different effect on SL, as well as total SIJ MRI SPARCC score in place of CRP, were tested in separate models. None of these variables, however, were found to be significant (see online supplemental table S3).

**Table 2:** Effect of socioeconomic factors and clinical variables on sick leave in separate models for ASDAS (main model), BASFI and BASMI

Multivariable model	Main model ASDAS	Model with a focus on BASFI	Model with a focus on BASMI
	HR (95% CI) (N=614)	HR (95% CI) (N=612)	HR (95% CI) (N=602)
*Explanatory variables*			
Age	1.05 (1.01 to 1.09)	1.04 (1.00 to 1.08)	1.04 (1.00 to 1.09)
Male gender	0.41 (0.20 to 0.86)	0.36 (0.18 to 0.75)	0.35 (0.15 to 0.82)
High education	0.48 (0.24 to 0.95)	0.37 (0.19 to 0.72)	0.42 (0.19 to 0.94)
ASDAS (CRP)	1.49 (1.04 to 2.13)	*	*
BASFI, 0–10	*	1.05 (0.91 to 1.23)	*
BASMI, 0–10	*	*	1.36 (0.96 to 1.93)
Smoking (current vs not)	2.55 (1.32 to 4.91)	2.57 (1.35 to 4.89)	2.71 (1.26 to 5.84)
Oral corticosteroid use (vs no)	–	3.00 (1.34 to 6.69)	–
TNFi use	2.41 (1.27 to 4.58)	2.07 (1.07 to 4.01)	2.55 (1.20 to 5.44)

The symbol '*' denotes that the specific variables were not tested in the models; the symbol '-' denotes that the specific variables were not significant in the models.

ASDAS, Ankylosing Spondylitis Disease Activity Score; BASFI, Bath Ankylosing Spondylitis Functional Index; BASMI, Bath Ankylosing Spondylitis Metrology Index; CRP, C reactive protein; TNFi, tumour necrosis factor inhibitor.

### Sensitivity analyses

Baseline characteristics of the ASAS criteria fulfilling subgroup of DESIR (n=423, 60%) are shown in online supplemental table S1 alongside the study population for comparison. In sensitivity analyses, similar findings were observed, with worse disease activity as measured by ASDAS associated with a higher hazard of SL (see online supplemental tables S2 and S3). Socioeconomic variables, in particular neither gender nor education, were associated with a higher hazard of SL, in this smaller group of patients (n=380) with a lower number of SL events (n=16).

## Discussion

This study, based on data from an early axSpA cohort of young, working-age individuals, reveals two main observations. First, that the incidence of SL in early axSpA is low and second, that both clinical and socioeconomic factors independently associate with SL. Specifically, across the study group, higher (university) education and male gender independently associated with a lower hazard of SL and older age and higher disease activity with higher hazard of SL. The findings suggest a role of socioeconomic factors, alongside active disease, in an important, yet generally poorly studied adverse work outcome: SL. The higher hazard of SL in those with higher disease activity even in the early stages of disease, suggests room for ‘intervention’ to prevent this adverse work outcome, especially in an era of biologics and other advanced therapies.

The association between education and SL is one that has been reported before, although in established disease and with education as a modifier of the association between disease activity or function and SL as opposed to an independent predictor.^17^ Specifically, higher disease activity or worse physical function were predictive of SL, but only in patients with low educational attainment.^17^ In our study, education and in particular, lower education was associated with a higher hazard of SL. This observation could be explained by residual confounding, whereby people with lower education are more likely to take manual jobs,^26^ which have further been shown to associate with poorer work outcome. Of note, a cross-sectional multicentre study showed that mastery, an important personal factor linked to self-efficacy, was associated with being employed in patients, but only in those with low education.^27^ A cross-sectional study identified lower educational status to be associated with the length of SL, but not with the likelihood of incurring any SL.^8^ Such associations can be challenging to interpret and one should keep in mind that it may not be the effect of education itself and that other work-related variables may be implicated in this path with SL as an outcome. Education could relate to type of job, self-management skills and coping ability,^17^ among other. Such insights are useful when considering an individual and their personal contextual factors along with disease characteristics, on outcomes relating to work ability.

Despite a consistency in our findings of worse disease activity relating to more SL with previous studies^12 15^ in axSpA, our study found no association between measures of function or mobility and the risk of SL. The latter differs from the findings of other studies, a likely explanation for this difference being the fact that our study was based in early axSpA with little amount of damage accrued over time, as we have previously shown.^26^ Furthermore, our study specifically aimed to address the impact of both socioeconomic and clinical factors on SL, recognising the potential relevance of socioeconomic factors alongside clinical factors, on this work outcome. This was reflected in our modelling approach, which placed the emphasis on both contextual and clinical factors on building the models. Our most parsimonious model retained individual personal contextual factors (age, gender, education) and smoking status, along with clinical disease parameters as previously identified in the literature. Smoking status in particular, featured as a strong contributor of the SL hazard, potentially in part also as a proxy for worse socioeconomic parameters. As with the observations for level of education, however, the possibility of residual confounding with other work-related factors implicated in the equation, cannot be excluded. In line with this observation, in previous analyses in DESIR we have seen smoking to be associated with MRI-SIJ inflammation specifically in patients with blue-collar jobs or low education.^26^ The retention of smoking in the models may have contributed for clinical variables like function and spinal mobility, not having an independent significant effect in the multivariable models, corrected for the above-mentioned socioeconomic factors, while they were significant in univariable analyses. Disentangling individual effects especially when it comes to complex relationships between disease, context and outcome can thus be challenging partly due to residual confounding which cannot always be controlled.^28^ Yet, in this regard, this study provides additional useful insights into our current understanding of disease, ‘context’ and outcome in axSpA. The effect of treatment and, in particular TNFi use, as seen in the multivariable analyses and the association with higher hazard of SL could represent confounding by indication. In other words, patients with worse disease were more likely to receive TNFi at some point during follow-up and were also at higher risk of SL. Additionally, it is also possible that TNFi were used in patients with a low chance of response to biologics, hence the association seen with SL.

Our study has several clinical implications, both from an individual as well as a societal perspective. The undertaking of SL is to a large extent a decision of the individual and could relate to underlying personal contextual factors. This may explain why educational status has been found in our study and in previous studies to be significantly associated with SL. The potential impact of this outcome on the health and social security system could also be substantial; in this respect, relevant education of both employees and employers but also rheumatologists, could play a role in guiding effective interventions to improve work ability and reduce SL. In addition, with previous reports suggesting a first episode of SL to predict future episodes of SL,^17^ this further highlights the need for addressing this adverse work outcome as part of an attempt both to optimise the existing care of patients but also to prevent future adverse work outcomes.

On the other hand, the contextual dependence of SL, could also represent a drawback. For example, individuals are likely to take more SL, if there is the financial safety net that allows them to do so. Social security systems and available support to employees varies across countries, thus limiting the generalisability of results. For example, in France individuals are entitled to paid SL, including extended SL, for serious or long illnesses, provided there is adequate supportive information. Paid SL can be up to 12 months, usually capped at 360 days within 3 years; however, paid SL can be granted for up to 3 years.

So, an important limitation of our study findings is the potential lack of generalisability of results. However, it is worth noting that, a study on SL across three nations showed no effect of country of residence on this outcome.^17^ Specifically, the risk of having an episode of SL did not depend on country. Second, patient responses for SL varied, as discussed in Methods section, with discrepancies noted in the way patients completed the relevant questions (through provision of a date or simply indicating whether on SL or not). Despite inconsistencies in the data, every effort was made to maximise and optimise the level of information available in this important work outcome, using various different sources of information from the DESIR CRF, including where available and appropriate, text information provided by patients. The identified inconsistencies in the data culminated in the decision to proceed with a safe, although restricted, approach of focusing on factors that associate with the first episode of SL.

Longitudinal data are necessary to look at potential factors that associate with SL, especially when they provide the possibility to study time-dependent variation of potential explanatory variables. In this regard, the DESIR cohort becomes uniquely relevant, especially in the study of socioeconomic factors which may have long-term influence on adverse work outcomes such as SL. We purposefully placed emphasis on the study of socioeconomic alongside clinical data, taking advantage of the recording of the former data in DESIR, which has led to the most parsimonious models being ones that indicate both socioeconomic and clinical factors to be contributory to SL. Furthermore, and as highlighted above, considering that most studies to date have focused on determinants of SL in established axSpA (r-axSpA),^8 12 15 17 29 30^ our study findings, focusing in early disease, provide new insights and enable the study of disease factors that associate with specific outcomes at the crucial, early stages of disease. Furthermore, the real-life, prospective nature of the cohort and the large patient sample allowing the detection of subtle associations between different factors of interest, in this case clinical and socioeconomic factors, present additional strenghts of this work.

In conclusion, this study has shown that the incidence of SL in this early axSpA cohort of young, working-age individuals is low. It also provides a deeper understanding of factors beyond clinical disease that associate with adverse work outcomes. The study suggests that, beyond the disease itself and its activity, socioeconomic factors associated with a potentially reversible adverse work outcome: SL. Thus, an appreciation of personal contextual factors and their potential independent association with SL, could allow the tailoring of care around individual needs and support people in their work role.

## Data Availability

Data are available upon reasonable request to the corresponding author and with approval from the DESIR committee.
