# Supplementary material for: Sick leave in early axial spondyloarthritis: the role of clinical and socioeconomic factors. Five-year data from the DESIR cohort
Source: RMD Open. 2021 Jun 25;7(2):e001685. doi: 10.1136/rmdopen-2021-001685 (PMC8237733; doi:10.1136/rmdopen-2021-001685)
Supplement: Supplementary data [file rmdopen-2021-001685supp001.pdf]

Supplementary File

Supplementary Table S1. Baseline characteristics of the study population and the ASAS criteria fulfilling subgroup.

BASELINE VARIABLES	Study population N=704 Mean (SD) or n, %	ASAS criteria subgroup N=423 Mean (SD) or n, %
Age, years	33.8 (8.6)	31.5 (7.3)
Male gender	324, 46%	223, 53%
Caucasian ethnicity	631, 90%	378, 89%
Higher education^	417, 59.4%	270, 64%
In employment	561, 80%	328, 78%
Blue-collar profession (of those employed)^^^^	96, 17%	64, 20%
Married/In couple^	445, 63%	257, 61%
Parental status, number of children^^		
- 0	294, 43%	204, 49.5%
- 1	133, 19%	81, 19.7%
- 2	170, 25%	95, 23.1%
- 3	68, 10%	25, 6.1%
- 4	11, 2%	4, 1.0%
- 5	7, 1%	2, 0.5%
- 6	1, 0%	1, 0.3%
Smoking, current^	256, 37%	167, 40%
HLA-B27 positivity^	409, 58%	371, 88%
Symptom duration, years^	1.5 (0.9)	1.6 (0.9)
ASDAS^^	2.7 (0.9)	2.6 (1.0)
Elevated CRP(>6mg/L)^^	194 (28.5)	135, 33%
CRP, mg/L^^	7.9 (13.6)	8.7 (13.9)

BASDAI, 0-10^	4.5 (2.0)	4.2 (2.0)
BASFI, 0-10^	3.0 (2.3)	2.9 (2.2)
BASMI, 0-10^^^	2.4 (1.0)	2.4 (0.9)
History of uveitis	65, 9%	43, 10%
History of psoriasis	117, 17%	63, 15%
History of IBD	35, 5%	20, 5%
History of peripheral arthritis^	49, 7%	28, 7%
NSAID score in last week, 0-400^^	55.9 (52.6)	61.6 (52.1)
TNFi use	0, 0%	0, 0%
Steroid use	85, 12%	59, 14%

*ASDAS, Ankylosing spondylitis (AS) disease activity score; CRP, C-Reactive Protein; BASDAI, Bath AS disease activity index; BASFI, Bath AS functional index; BASMI, Bath AS Metrology Index; IBD, inflammatory bowel disease; NSAIDs, non-steroidal anti-inflammatory drugs; TNFi, Tumour Necrosis Factor inhibitor. *Indicates variable n where total n not available due to missing data. Missing data: ^<1% missing; ^^<5% missing; ^^^<10% missing; ^^^^<15% missing.*

Supplementary Table S2. Univariable associations between independent variables and sick leave in the study population and the ASAS fulfilling subgroup.

	**Study population**	**ASAS criteria subgroup**
	**Sick Leave (SL)**	**Sick Leave (SL)**
	HR (95% CI)	HR (95% CI)
Independent variable		
Age, years	1.04 (1.01, 1.08)**	1.02 (0.96, 1.09)
Male gender^	0.37 (0.19, 0.74)**	0.34 (0.12, 0.98)**
Caucasian (vs all other)	0.69 (0.27, 1.75)	0.45 (0.13, 1.56)
Higher education (vs lower)	0.33 (0.17, 0.61)***	0.52 (0.20, 1.34)*
Blue-collar (vs white)	1.15 (0.50, 2.63)	1.22 (0.34, 4.45)
Married (vs not)	1.58 (0.78, 3.21)	3.73 (0.85, 16.38)*
Parental status, number of children	1.05 (0.82, 1.35)	1.09 (0.72, 1.65)
Smoking (vs not)	2.40 (1.31, 4.37)***	4.34 (1.53, 12.35)**
HLA-B27 positive^	0.51 (0.28, 0.93)**	2.05 (0.27, 15.5)
Symptom duration, years^	1.14 (0.82, 1.59)	0.94 (0.55, 1.60)
ASDAS (CRP)	1.83 (1.34, 2.50)***	2.37 (1.45, 3.88)***
CRP abnormal (>6mg/L)at visit (vs not)	0.99 (0.95, 1.03)	1.00 (0.95, 1.05)
BASDAI, 0-10	1.34 (1.17, 1.53)***	1.53 (1.23, 1.91)***
BASFI, 0-10	1.24 (1.09, 1.40)***	1.41 (1.16, 1.72)***
BASMI, 0-10	1.76 (1.31, 2.38)***	1.20 (0.62, 2.29)
Presence of uveitis	0.58 (0.18, 1.87)	1.40 (0.40, 4.93)
Presence of psoriasis	0.80 (0.37, 1.73)	0.82 (0.23, 2.87)
Presence of IBD	0.59 (0.14, 2.46)	+
Presence of peripheral arthritis	1.38 (0.33, 5.73)	+
Hip involvement (baseline) vs not^	1.15 (0.51, 2.60)	0.89 (0.20, 3.88)
Comorbidity count, 0-4[#]	1.77 (1.22, 2.57)***	1.64 (0.87, 3.07)*
Radiographic change (mNY positive vs negative)	0.60 (0.21, 1.67)	1.07 (0.35, 3.28)

MRI-SIJ inflammation (positive vs negative)	0.66 (0.31, 1.44)	0.68 (0.24, 1.95)
NSAID use last 6m (vs no use)	1.01 (1.00, 1.01)*	1.01 (1.00, 1.03)**
NSAID score last week, 0-400	1.01 (1.00, 1.01)*	1.01 (1.00, 1.02)*
Oral Corticosteroid use (vs no)	3.90 (1.80, 8.46)***	5.18(1.68, 15.96)***
csDMARD use last 6m (vs no)	1.25 (0.44, 3.59)	1.00 (0.12, 7.97)
TNFi use	2.86 (1.55, 5.28)***	2.70 (1.02, 7.14)**

***p<0.005, **p<0.05, *p<0.2; ^Variables only at baseline. #Comorbidity count includes: chronic pulmonary disease, ischaemic heart disease, pericarditis, heart failure, cardiac valve disease including aortic insufficiency, heart rhythm disorders, hypertension, cerebrovascular accidents, diabetes, gastric ulcers/perforation/haemorrhage, depression/anxiety using SF36_MCS (threshold of ≤38 to identify the presence of either depression or anxiety), lymphoproliferative disease, organ neoplastic disease. Higher education refers to university education (compared to lower, in this study: primary or secondary education). + Models failed to converge due to lack of enough failures in each category. *ASDAS, Ankylosing Spondylitis disease activity score; CRP, C-Reactive Protein; BASDAI, Bath AS disease activity index; BASFI, Bath AS functional index; BASMI, Bath AS Metrology Index; IBD, inflammatory bowel disease; MIR-SIJ, Magnetic Resonance Imaging-Sacroiliac Joints; NSAID, non-steroidal anti-inflammatory drugs; TNFi, Tumour Necrosis Factor inhibitor; MRI, Magnetic Resonance Imaging; csDMARD, conventional synthetic Disease-modifying anti-rheumatic drug.*

Table S3. Additional multivariable models with sick leave as outcome in separate models for BASDAI, BASDAI/CRP and MRI SIJ inflammation based on SPARCC, included as independent variables.

Multivariable model	Focus on BASDAI	Focus on BASDAI & CRP	Focus on MRI SIJ inflammation (SPARCC)
	HR **(95% CI) (N = 617)**	HR **(95% CI) (N = 614)**	HR **(95% CI) (N = 597)**
Explanatory variables			
Age	1.03 (1.00, 1.07)	1.0 (1.00, 1.08)	1.04 (1.00, 1.08)
Male gender	0.39 (0.18, 0.81)	0.41 (0.20, 0.87)	0.38 (0.17, 0.84)
High education	0.41 (0.21, 0.80)	0.46 (0.23, 0.90)	0.46 (0.23, 0.90)
BASDAI, 0-10	1.16 (1.00, 1.35)	1.16 (0.99, 1.35)	1.18 (1.01, 1.37)
CRP (mg/L)	-	0.99 (0.95, 1.04)	-
Smoking (current vs not)	2.54 (1.34, 4.83)	2.58 (1.34, 4.99)	2.30 (1.19, 4.46)
TNFi use	2.30 (1.23, 4.31)	2.32 (1.22, 4.43)	2.37 (1.25, 4.52)
SIJ MRI, SPARCC score, 0-72			1.02 (0.97, 1.08)

CRP, C-Reactive Protein; BASDAI, Bath AS disease activity index; TNFi, Tumour Necrosis Factor inhibitor; SIJ, Sacroiliac joints; MRI, Magnetic Resonance Imaging; SPARCC, Spondyloarthritis Research Consortium of Canada scoring system.

Supplementary Figure S1. Time to sick leave in fully adjusted model, based on educational level (primary/secondary [low] versus university high]).

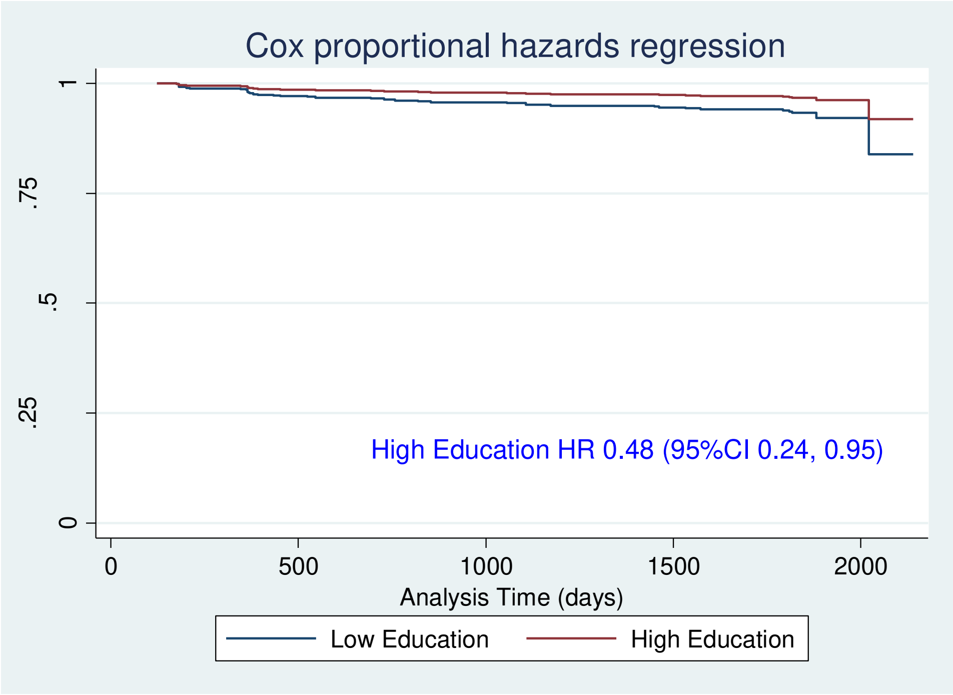

Model adjusted for: age, gender, disease activity based on Ankylosing Spondylitis disease activity score using C-reactive protein (ASDAS-CRP), smoking and use of Tumour Necrosis Factor inhibitors (TNFi) (Main Model, Table 2).
